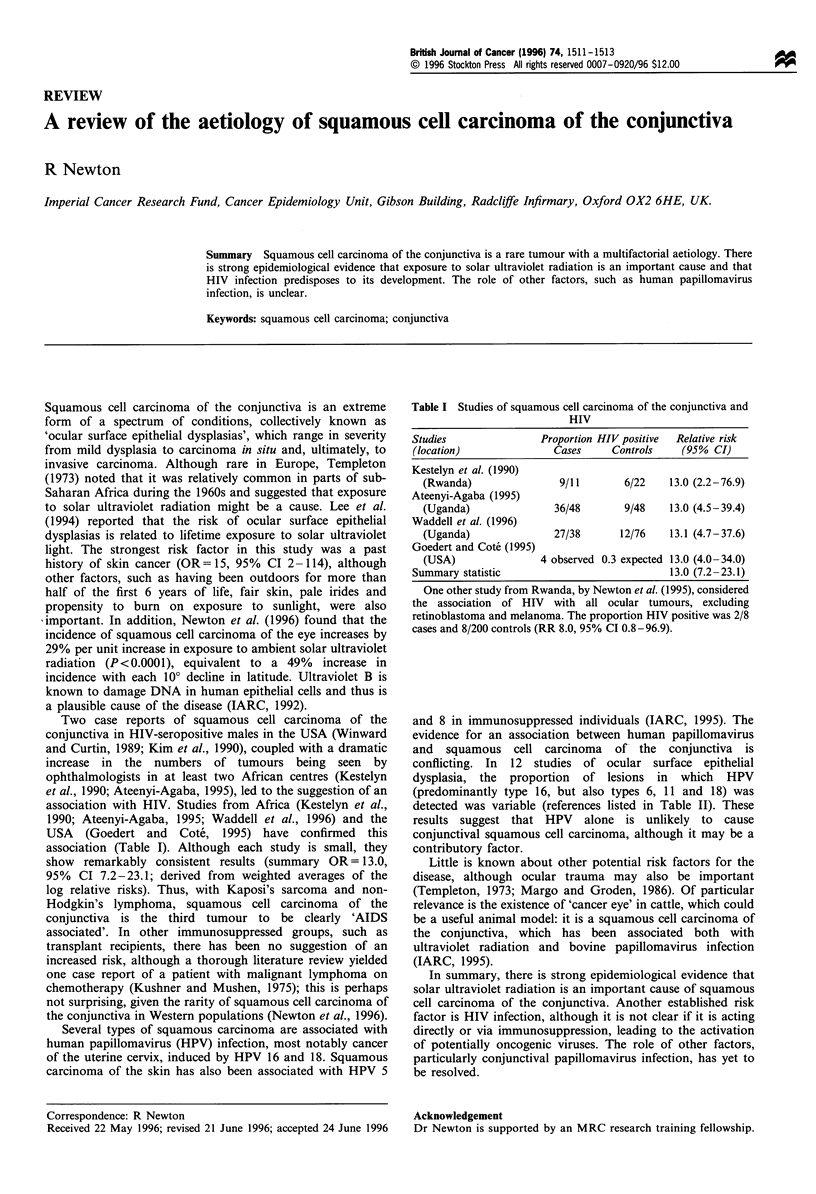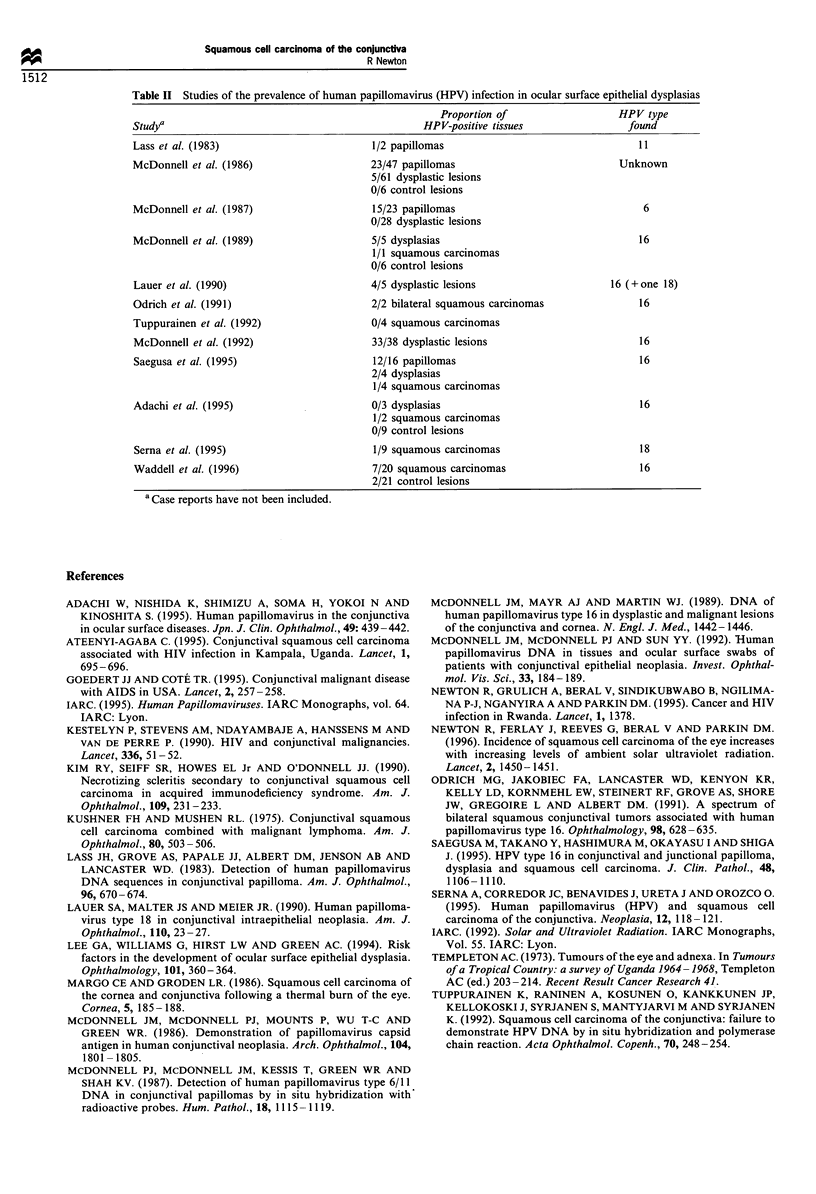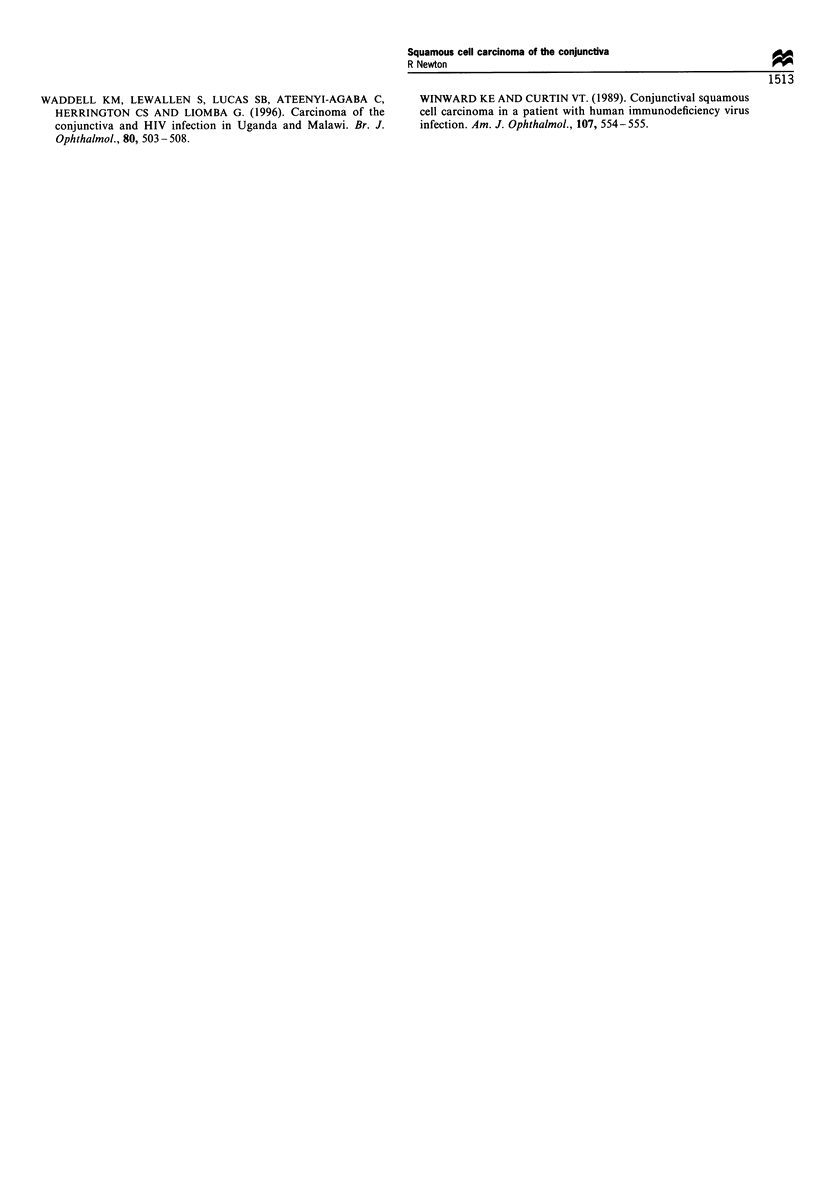# A review of the aetiology of squamous cell carcinoma of the conjunctiva.

**DOI:** 10.1038/bjc.1996.581

**Published:** 1996-11

**Authors:** R. Newton

**Affiliations:** Imperial Cancer Research Fund, Cancer Epidemiology Unit, Radcliffe Infirmary, Oxford, UK.

## Abstract

Squamous cell carcinoma of the conjunctiva is a rare tumour with a multifactorial aetiology. There is strong epidemiological evidence that exposure to solar ultraviolet radiation is an important cause and that HIV infection predisposes to its development. The role of other factors, such as human papillomavirus infection, is unclear.


British Journal of Cancer (1996) 74, 1511-1513
© 1996 Stockton Press All rights reserved 0007-0920/96 $12.00

REVIEW

### A review of the aetiology of squamous cell carcinoma of the conjunctiva

R Newton

*Imperial Cancer Research Fund, Cancer Epidemiology Unit, Gibson Building, Radcliffe Infirmary, Oxford OX2 6HE, UK.*

**Summary** Squamous cell carcinoma of the conjunctiva is a rare tumour with a multifactorial aetiology. There is strong epidemiological evidence that exposure to solar ultraviolet radiation is an important cause and that HIV infection predisposes to its development. The role of other factors, such as human papillomavirus infection, is unclear.

**Keywords:** squamous cell carcinoma; conjunctiva

Squamous cell carcinoma of the conjunctiva is an extreme form of a spectrum of conditions, collectively known as 'ocular surface epithelial dysplasias', which range in severity from mild dysplasia to carcinoma *in situ* and, ultimately, to invasive carcinoma. Although rare in Europe, Templeton (1973) noted that it was relatively common in parts of sub-Saharan Africa during the 1960s and suggested that exposure to solar ultraviolet radiation might be a cause. Lee *et al.* (1994) reported that the risk of ocular surface epithelial dysplasias is related to lifetime exposure to solar ultraviolet light. The strongest risk factor in this study was a past history of skin cancer (OR = 15, 95% CI 2-114), although other factors, such as having been outdoors for more than half of the first 6 years of life, fair skin, pale irides and propensity to burn on exposure to sunlight, were also important. In addition, Newton *et al.* (1996) found that the incidence of squamous cell carcinoma of the eye increases by 29% per unit increase in exposure to ambient solar ultraviolet radiation ($P<0.0001$), equivalent to a 49% increase in incidence with each 10° decline in latitude. Ultraviolet B is known to damage DNA in human epithelial cells and thus is a plausible cause of the disease (IARC, 1992).

Two case reports of squamous cell carcinoma of the conjunctiva in HIV-seropositive males in the USA (Winward and Curtin, 1989; Kim *et al.*, 1990), coupled with a dramatic increase in the numbers of tumours being seen by ophthalmologists in at least two African centres (Kestelyn *et al.*, 1990; Ateenyi-Agaba, 1995), led to the suggestion of an association with HIV. Studies from Africa (Kestelyn *et al.*, 1990; Ateenyi-Agaba, 1995; Waddell *et al.*, 1996) and the USA (Goedert and Coté, 1995) have confirmed this association (Table I). Although each study is small, they show remarkably consistent results (summary OR = 13.0, 95% CI 7.2-23.1; derived from weighted averages of the log relative risks). Thus, with Kaposi's sarcoma and non-Hodgkin's lymphoma, squamous cell carcinoma of the conjunctiva is the third tumour to be clearly 'AIDS associated'. In other immunosuppressed groups, such as transplant recipients, there has been no suggestion of an increased risk, although a thorough literature review yielded one case report of a patient with malignant lymphoma on chemotherapy (Kushner and Mushen, 1975); this is perhaps not surprising, given the rarity of squamous cell carcinoma of the conjunctiva in Western populations (Newton *et al.*, 1996).

Several types of squamous carcinoma are associated with human papillomavirus (HPV) infection, most notably cancer of the uterine cervix, induced by HPV 16 and 18. Squamous carcinoma of the skin has also been associated with HPV 5 and 8 in immunosuppressed individuals (IARC, 1995). The evidence for an association between human papillomavirus and squamous cell carcinoma of the conjunctiva is conflicting. In 12 studies of ocular surface epithelial dysplasia, the proportion of lesions in which HPV (predominantly type 16, but also types 6, 11 and 18) was detected was variable (references listed in Table II). These results suggest that HPV alone is unlikely to cause conjunctival squamous cell carcinoma, although it may be a contributory factor.

**Table I** Studies of squamous cell carcinoma of the conjunctiva and HIV

| *Studies (location)* | *Proportion HIV positive Cases* | *Controls* | *Relative risk (95% CI)* |
|---|---|---|---|
| Kestelyn *et al.* (1990) (Rwanda) | 9/11 | 6/22 | 13.0 (2.2-76.9) |
| Ateenyi-Agaba (1995) (Uganda) | 36/48 | 9/48 | 13.0 (4.5-39.4) |
| Waddell *et al.* (1996) (Uganda) | 27/38 | 12/76 | 13.1 (4.7-37.6) |
| Goedert and Coté (1995) (USA) | 4 observed | 0.3 expected | 13.0 (4.0-34.0) |
| Summary statistic | | | 13.0 (7.2-23.1) |

One other study from Rwanda, by Newton *et al.* (1995), considered the association of HIV with all ocular tumours, excluding retinoblastoma and melanoma. The proportion HIV positive was 2/8 cases and 8/200 controls (RR 8.0, 95% CI 0.8-96.9).

Little is known about other potential risk factors for the disease, although ocular trauma may also be important (Templeton, 1973; Margo and Groden, 1986). Of particular relevance is the existence of 'cancer eye' in cattle, which could be a useful animal model: it is a squamous cell carcinoma of the conjunctiva, which has been associated both with ultraviolet radiation and bovine papillomavirus infection (IARC, 1995).

In summary, there is strong epidemiological evidence that solar ultraviolet radiation is an important cause of squamous cell carcinoma of the conjunctiva. Another established risk factor is HIV infection, although it is not clear if it is acting directly or via immunosuppression, leading to the activation of potentially oncogenic viruses. The role of other factors, particularly conjunctival papillomavirus infection, has yet to be resolved.

Correspondence: R Newton
Received 22 May 1996; revised 21 June 1996; accepted 24 June 1996

**Acknowledgement**

Dr Newton is supported by an MRC research training fellowship.

Table II Studies of the prevalence of human papillomavirus (HPV) infection in ocular surface epithelial dysplasias

| Study[a] | Proportion of HPV-positive tissues | HPV type found |
|---|---|---|
| Lass *et al.* (1983) | 1/2 papillomas | 11 |
| McDonnell *et al.* (1986) | 23/47 papillomas<br>5/61 dysplastic lesions<br>0/6 control lesions | Unknown |
| McDonnell *et al.* (1987) | 15/23 papillomas<br>0/28 dysplastic lesions | 6 |
| McDonnell *et al.* (1989) | 5/5 dysplasias<br>1/1 squamous carcinomas<br>0/6 control lesions | 16 |
| Lauer *et al.* (1990) | 4/5 dysplastic lesions | 16 (+one 18) |
| Odrich *et al.* (1991) | 2/2 bilateral squamous carcinomas | 16 |
| Tuppurainen *et al.* (1992) | 0/4 squamous carcinomas | |
| McDonnell *et al.* (1992) | 33/38 dysplastic lesions | 16 |
| Saegusa *et al.* (1995) | 12/16 papillomas<br>2/4 dysplasias<br>1/4 squamous carcinomas | 16 |
| Adachi *et al.* (1995) | 0/3 dysplasias<br>1/2 squamous carcinomas<br>0/9 control lesions | 16 |
| Serna *et al.* (1995) | 1/9 squamous carcinomas | 18 |
| Waddell *et al.* (1996) | 7/20 squamous carcinomas<br>2/21 control lesions | 16 |

[a] Case reports have not been included.

### References

ADACHI W, NISHIDA K, SHIMIZU A, SOMA H, YOKOI N AND KINOSHITA S. (1995). Human papillomavirus in the conjunctiva in ocular surface diseases. *Jpn. J. Clin. Ophthalmol.*, **49:** 439–442.

ATEENYI-AGABA C. (1995). Conjunctival squamous cell carcinoma associated with HIV infection in Kampala, Uganda. *Lancet*, **1**, 695–696.

GOEDERT JJ AND COTÉ TR. (1995). Conjunctival malignant disease with AIDS in USA. *Lancet*, **2**, 257–258.

IARC. (1995). *Human Papillomaviruses*. IARC Monographs, vol. 64. IARC: Lyon.

KESTELYN P, STEVENS AM, NDAYAMBAJE A, HANSSENS M AND VAN DE PERRE P. (1990). HIV and conjunctival malignancies. *Lancet*, **336**, 51–52.

KIM RY, SEIFF SR, HOWES EL Jr AND O'DONNELL JJ. (1990). Necrotizing scleritis secondary to conjunctival squamous cell carcinoma in acquired immunodeficiency syndrome. *Am. J. Ophthalmol.*, **109**, 231–233.

KUSHNER FH AND MUSHEN RL. (1975). Conjunctival squamous cell carcinoma combined with malignant lymphoma. *Am. J. Ophthalmol.*, **80**, 503–506.

LASS JH, GROVE AS, PAPALE JJ, ALBERT DM, JENSON AB AND LANCASTER WD. (1983). Detection of human papillomavirus DNA sequences in conjunctival papilloma. *Am. J. Ophthalmol.*, **96**, 670–674.

LAUER SA, MALTER JS AND MEIER JR. (1990). Human papillomavirus type 18 in conjunctival intraepithelial neoplasia. *Am. J. Ophthalmol.*, **110**, 23–27.

LEE GA, WILLIAMS G, HIRST LW AND GREEN AC. (1994). Risk factors in the development of ocular surface epithelial dysplasia. *Ophthalmology*, **101**, 360–364.

MARGO CE AND GRODEN LR. (1986). Squamous cell carcinoma of the cornea and conjunctiva following a thermal burn of the eye. *Cornea*, **5**, 185–188.

MCDONNELL JM, MCDONNELL PJ, MOUNTS P, WU T-C AND GREEN WR. (1986). Demonstration of papillomavirus capsid antigen in human conjunctival neoplasia. *Arch. Ophthalmol.*, **104**, 1801–1805.

MCDONNELL PJ, MCDONNELL JM, KESSIS T, GREEN WR AND SHAH KV. (1987). Detection of human papillomavirus type 6/11 DNA in conjunctival papillomas by in situ hybridization with radioactive probes. *Hum. Pathol.*, **18**, 1115–1119.

MCDONNELL JM, MAYR AJ AND MARTIN WJ. (1989). DNA of human papillomavirus type 16 in dysplastic and malignant lesions of the conjunctiva and cornea. *N. Engl. J. Med.*, 1442–1446.

MCDONNELL JM, MCDONNELL PJ AND SUN YY. (1992). Human papillomavirus DNA in tissues and ocular surface swabs of patients with conjunctival epithelial neoplasia. *Invest. Ophthalmol. Vis. Sci.*, **33**, 184–189.

NEWTON R, GRULICH A, BERAL V, SINDIKUBWABO B, NGILIMANA P-J, NGANYIRA A AND PARKIN DM. (1995). Cancer and HIV infection in Rwanda. *Lancet*, **1**, 1378.

NEWTON R, FERLAY J, REEVES G, BERAL V AND PARKIN DM. (1996). Incidence of squamous cell carcinoma of the eye increases with increasing levels of ambient solar ultraviolet radiation. *Lancet*, **2**, 1450–1451.

ODRICH MG, JAKOBIEC FA, LANCASTER WD, KENYON KR, KELLY LD, KORNMEHL EW, STEINERT RF, GROVE AS, SHORE JW, GREGOIRE L AND ALBERT DM. (1991). A spectrum of bilateral squamous conjunctival tumors associated with human papillomavirus type 16. *Ophthalmology*, **98**, 628–635.

SAEGUSA M, TAKANO Y, HASHIMURA M, OKAYASU I AND SHIGA J. (1995). HPV type 16 in conjunctival and junctional papilloma, dysplasia and squamous cell carcinoma. *J. Clin. Pathol.*, **48**, 1106–1110.

SERNA A, CORREDOR JC, BENAVIDES J, URETA J AND OROZCO O. (1995). Human papillomavirus (HPV) and squamous cell carcinoma of the conjunctiva. *Neoplasia*, **12**, 118–121.

IARC. (1992). *Solar and Ultraviolet Radiation*. IARC Monographs, Vol. 55. IARC: Lyon.

TEMPLETON AC. (1973). Tumours of the eye and adnexa. In *Tumours of a Tropical Country: a survey of Uganda 1964–1968*, Templeton AC (ed.) 203–214. *Recent Result Cancer Research 41.*

TUPPURAINEN K, RANINEN A, KOSUNEN O, KANKKUNEN JP, KELLOKOSKI J, SYRJANEN S, MANTYJARVI M AND SYRJANEN K. (1992). Squamous cell carcinoma of the conjunctiva: failure to demonstrate HPV DNA by in situ hybridization and polymerase chain reaction. *Acta Ophthalmol. Copenh.*, **70**, 248–254.

WADDELL KM, LEWALLEN S, LUCAS SB, ATEENYI-AGABA C, HERRINGTON CS AND LIOMBA G. (1996). Carcinoma of the conjunctiva and HIV infection in Uganda and Malawi. *Br. J. Ophthalmol.*, **80,** 503 – 508.

WINWARD KE AND CURTIN VT. (1989). Conjunctival squamous cell carcinoma in a patient with human immunodeficiency virus infection. *Am. J. Ophthalmol.*, **107,** 554 – 555.